# A Bacterial Symbiont Protects Honey Bees from Fungal Disease

**DOI:** 10.1128/mBio.00503-21

**Published:** 2021-06-08

**Authors:** Delaney L. Miller, Eric A. Smith, Irene L. G. Newton

**Affiliations:** a Department of Biology, Indiana University, Bloomington, Indiana, USA; University of Connecticut

**Keywords:** acetic acid microbes, *Parasaccharibacter apium*, *Bombella apis*, symbiosis, *Apis mellifera*, *Bombella*, *Nosema*, symbiosis

## Abstract

Fungal pathogens, among other stressors, negatively impact the productivity and population size of honey bees, one of our most important pollinators (1, 2), in particular their brood (larvae and pupae) (3, 4). Understanding the factors that influence disease incidence and prevalence in brood may help us improve colony health and productivity. Here, we examined the capacity of a honey bee-associated bacterium, Bombella apis, to suppress the growth of fungal pathogens and ultimately protect bee brood from infection. Our results showed that strains of *B. apis* inhibit the growth of two insect fungal pathogens, Beauveria bassiana and Aspergillus flavus, *in vitro*. This phenotype was recapitulated *in vivo*; bee broods supplemented with *B. apis* were significantly less likely to be infected by A. flavus. Additionally, the presence of *B. apis* reduced sporulation of A. flavus in the few bees that were infected. Analyses of biosynthetic gene clusters across *B. apis* strains suggest antifungal candidates, including a type 1 polyketide, terpene, and aryl polyene. Secreted metabolites from *B. apis* alone were sufficient to suppress fungal growth, supporting the hypothesis that fungal inhibition is mediated by an antifungal metabolite. Together, these data suggest that *B. apis* can suppress fungal infections in bee brood via secretion of an antifungal metabolite.

## INTRODUCTION

Emerging fungal pathogens pose major threats to animal and plant populations (5), and as the climate begins to warm, it is predicted that we will see an increase in fungal pathogens (10). Among insects, fungal pathogens are currently the most common causal agent of disease and historically have plagued insect hosts for over 300 million years (8, 11). Therefore, insects have had to develop strategies to combat these fungal pathogens through their immune responses, behavioral modifications, and, in some cases, microbial symbiosis (12–14). The honey bee is an excellent model in which to investigate fungus-host-symbiont interactions. The honey bee worker interacts with environmental microbes through foraging, bringing pollen, nectar, and associated microbes back to the colony. In recent years, drastic population declines in honey bees (2) have been reported as the result of a combination of stressors, including or leading to opportunistic fungal infections (1, 4, 15). Importantly, within the colony, the most susceptible individuals are arguably the bee brood (larvae and pupae), which are exposed to fungal pathogens, notably chalkbrood (*Ascophaera apis*) and stonebrood (Aspergillus flavus) (3, 4), but cannot groom spores from their bodies. Although the spread of fungal disease among the brood can be limited by the hygienic behavior of honey bee nurses (16), it does not prevent the establishment of infection.

Honey bee broods are reared on a larval diet that is colonized by a few bacterial taxa (Bombella apis, *Lactobacillus kunkeii*, *Fructobacillus* spp., and, infrequently, *Bifidobacterium* spp.) (17, 18). We hypothesized that these microbes play a defensive role in honey bee brood development. In honey bee workers, which harbor a different microbiota than brood, susceptibility to various pathogens correlates with changes in their microbiome composition and abundance (19–26). In aggregate, this suggests that honey bee-associated microbiota have profound impacts on disease outcomes, which may be mediated by the presence or absence of key players. By extension, it is possible that brood-associated microbes play a similar defensive role.

One of the most prevalent brood-associated microbes is *B. apis* (formerly *Parasaccharibacter apium*) (27), an acetic-acid bacterium found in association with nectar and royal jelly. Within the colony it is distributed across niches, including larvae, the queen’s gut, worker hypopharyngeal glands, and nectar stores (28, 29). Many of the niches it colonizes, particularly the larvae, are susceptible to fungal infection and/or contamination, and its localization to these niches may be indicative of a protective role. Furthermore, in honey bee adults, increased *B. apis* load is negatively correlated with *Nosema* prevalence (a fungal pathogen of adults), suggesting interactive effects (23). Importantly, since *B. apis* is not found in appreciable numbers within adult guts, this effect may be the result of indirect influences on honey bee adult health.

Here, we examined the potential for *B. apis* to inhibit fungal establishment both *in vitro* and *in vivo*, using infection assays in laboratory-reared broods. Furthermore, we show that *B. apis* secretes an antifungal metabolite by using assays with spent medium from *B. apis* and predicting antifungal gene clusters in the *B. apis* genome with antiSMASH ((antibiotic and Secondary Metabolite Analysis Shell) (30). To determine the impact of *B. apis* on fungal colonization, we used two different insect pathogens in our assays: Beauveria bassiana, a generalist pathogen that infects 70% of insect species, and A. flavus, an opportunistic pathogen of honey bee brood. While not a known pathogen of honey bees, B. bassiana is a well-characterized model for entomopathogenic fungi and has been suggested as a biocontrol agent for the bee parasite, varroa mites. A. flavus, on the other hand, is the most pathogenic species of the Aspergillus spp., which are routinely found in approximately 81% of late-instar larvae (4). Our results identify a significant and dramatic reduction in fungal infection, provided by the *B. apis*-secreted metabolite, to honey bee brood, identifying a previously unknown protective symbiosis in the honey bee.

## RESULTS

**FIG 1:**
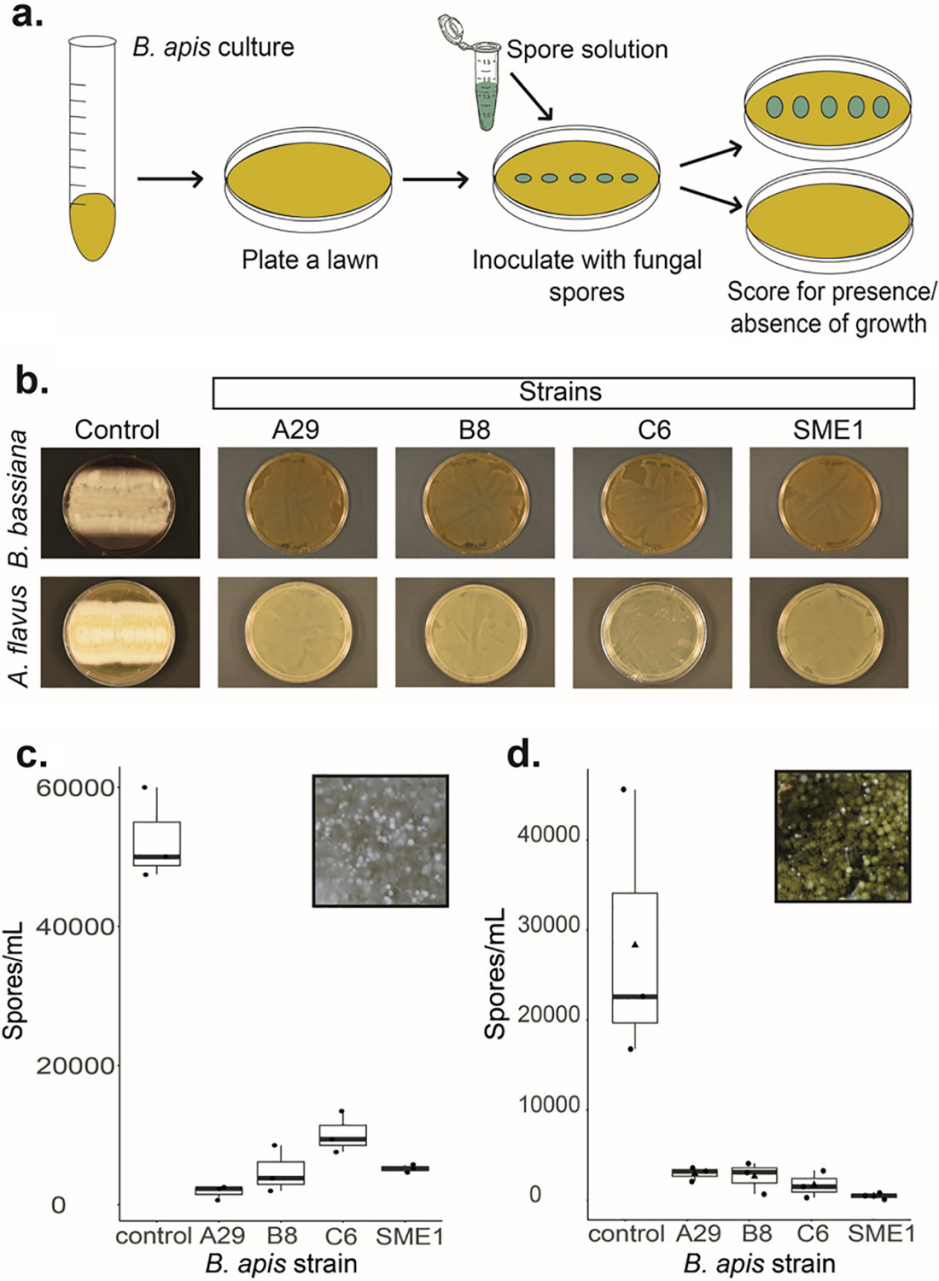
*B. apis* outcompetes fungal pathogens *in vitro.* (a) The ability of each fungal isolate to grow in the presence of *B. apis* was qualitatively assayed by plating 10^3^ spores of each isolate across a lawn of *B. apis*. (b) Compared to controls of 10^3^ spores plated on fresh media, the presence of *B. apis* completely inhibited fungal growth. (c) When cocultured in liquid media, the presence of *B. apis* significantly reduced the number of spores produced by B. bassiana (Kruskal-Wallis; χ^2^ = 11.7, df = 4, *P* = 0.01973; pairwise comparisons to control A29, *t* = 13.114, df = 2.0996, *P* = 0.019056; B8, *t* = 11.147, df = 2.9658, *P* = 0.00652; C6, *t* = 10.121, df = 2.7744, *P* = 0.011404; SME1, *t* = 12.352, df = 2.0277, *P* = 0.024652). (d) *B. apis* also significantly reduced the number of spores produced by A. flavus (Kruskal-Wallis; χ^2^  = 9.9, df = 4, *P*  = 0.04215); however, pairwise comparisons between control and SM from each strain were not significantly different due to the variation in the control samples. To control for nutritional effects, in panels c and d, experimental wells contained the same volume of fresh media as the controls in addition to *B. apis* culture. Each experimental group consists of three biological replicates. Sporulation was quantified for each well via hemocytometer.

To determine the ability of *B. apis* to inhibit fungal growth *in vitro*, we competed each fungal pathogen with one of four *B. apis* strains, isolated from apiaries in the United States (Fig. 1a; see also Fig. S1 in the supplemental material). In the presence of *B. apis* strains, fungal growth was either suppressed or completely inhibited (Fig. 1b). The presence of *B. apis* significantly inhibited the growth of B. bassiana and A. flavus. Quantitatively, the presence of *B. apis* also reduced sporulation of both pathogens. To quantify fungal inhibition, we counted spores of B. bassiana or A. flavus cocultured with *B. apis*. The number of spores produced by both B. bassiana and A. flavus was reduced by an order of magnitude, on average (Fig. 1c), compared to controls without the bacteria, showing that *B. apis* can suppress growth of both pathogens.

10.1128/mBio.00503-21.1 FIG S1 (A) Maximum-likelihood 16S rRNA gene sequence tree for strains used in this study. Saccharibacter floricola and Gluconobacter oxydans were used as outgroups. Sequences were downloaded from GenBank and aligned with the SINA aligner. The tree was constructed with RAxML and visualized with FigTree. Numbers at nodes represent bootstrap support from 1,000 bootstrap pseudoreplicates. (B) Core-ortholog maximum-likelihood phylogeny. All genomes were downloaded from GenBank, and core orthologs were identified using OrthoMCL. Alignments of core orthologs were made using MAFFT and concatenated together. As described for panel A, the tree was constructed with RAxML and visualized with FigTree. Numbers at nodes represent bootstrap support from 1,000 bootstrap pseudoreplicates. Download FIG S1, EPS file, 0.9 MB. Copyright © 2021 Miller et al. 2021 Miller et al. https://creativecommons.org/licenses/by/4.0/ This content is distributed under the terms of the Creative Commons Attribution 4.0 International license.

**FIG 2:**
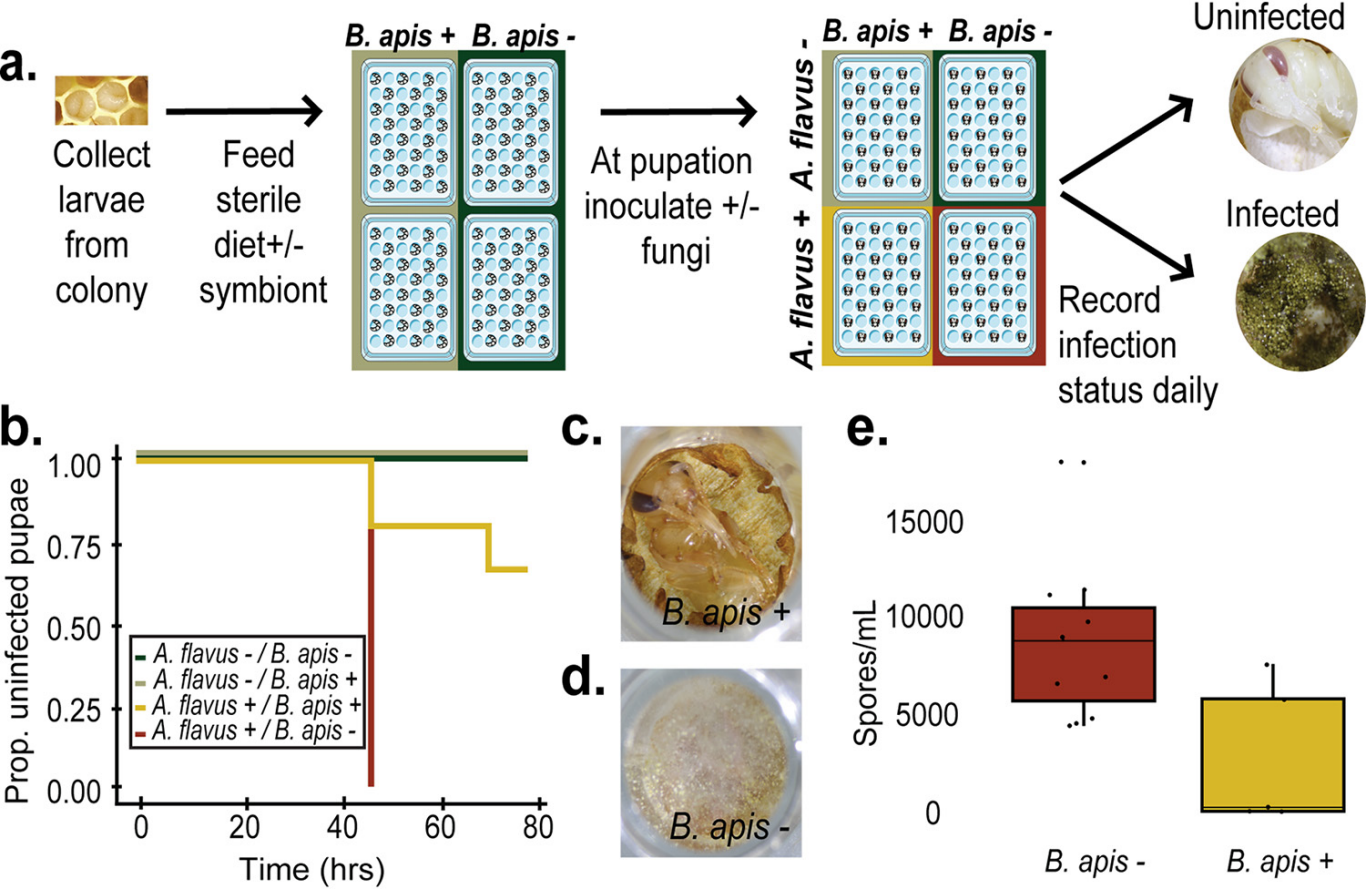
Bee broods supplemented with *B. apis* are less susceptible to infection with A. flavus. (a) First-instar larvae (*n* = 45) collected from the apiary were reared on sterile larval diet with or without *B. apis* (AJP2). Five days after pupation, each pupa was inoculated with 10^3^ spores of A. flavus with or without *B. apis* or carrier (0.01% Triton X-100) as a control (without A. flavus/without *B. apis*, *n* = 8; without A. flavus*/*with *B. apis*, *n* = 11; with A. flavus*/*without *B. apis*, *n* = 11; without A. flavus*/*with *B. apis*, *n* = 15). (b) Of the pupae inoculated with A. flavus, those without *B. apis* all showed signs of infection by 48 h, whereas 66% of those with *B. apis* never developed infections (χ^2^ = 14.8, df  = 1, *P* < 0.001). (c and d) Representative photos of individual pupae 48 h postinfection show the prominent difference in infection between the two groups. (e) Pupae with *B. apis* that did become infected (4 out of 15) had lower intensity infections, producing significantly (*t* = 5.5052, df = 5.5751, *P* = 0.0019) fewer spores than those without *B. apis*. The same outcome was replicated in three separate experiments, including with a different *B. apis* strain (Fig. S2).

To test if *B. apis* can prevent fungal infections *in vivo*, we collected larvae from our apiary and reared them on larval diet (as defined in Schmehl et al. [47]) supplemented with either *B. apis* or a sterile medium control. Once reared to pupae, the cohort was inoculated with A. flavus or a sterile MRS medium control, and the presence of infection was scored until adulthood (Fig. 2a). Pupae that were supplemented with *B. apis* (strain AJP2) as larvae were significantly more likely to resist fungal infection (χ^2^ = 14.8, df = 1, *P* < 0.001), with 66% of the cohort surviving to adulthood with no signs of infection (Fig. 2b and c). In sharp contrast, without *B. apis*, no pupae survived to adulthood (Fig. 2b and d). Interestingly, in the 34% of *B. apis*-supplemented pupae that succumbed to fungal infection, the number of spores produced was 68% lower than that of the control (MRS medium-supplemented pupae) on average (Fig. 2e; *t* = 2.9116, df = 8.4595, *P* = 0.01842). Taken together, these results suggest that the presence of *B. apis* increases the host’s likelihood of survival under fungal challenge while decreasing the pathogen’s spore load and potential to spread infection to new hosts. This experiment was repeated with another *B. apis* strain with the same significant result (Fig. S2).

10.1128/mBio.00503-21.2 FIG S2 Bee brood are protected from fungal infection, independent of *B. apis* strain identity. (a) First-instar larvae (*n* = 20) collected from the apiary were reared on sterile larval diet with or without *B*. *apis* (A29). Five days after pupation, each pupa was inoculated with 10^3^ spores of A. flavus with or without *B. apis* or 0.01% Triton X-100 as a control. Pupae supplemented with A29 were more likely to survive to adulthood (χ^2^ = 3.4, df = 1, *P* = 0.07) (b) Presence of *B. apis* (A29) significantly reduced (*t* = 5.5052, df = 5.5751, *P* = 0.001914) sporulation in infected pupae. Download FIG S2, EPS file, 0.7 MB. Copyright © 2021 Miller et al. 2021 Miller et al. https://creativecommons.org/licenses/by/4.0/ This content is distributed under the terms of the Creative Commons Attribution 4.0 International license.

**FIG 3:**
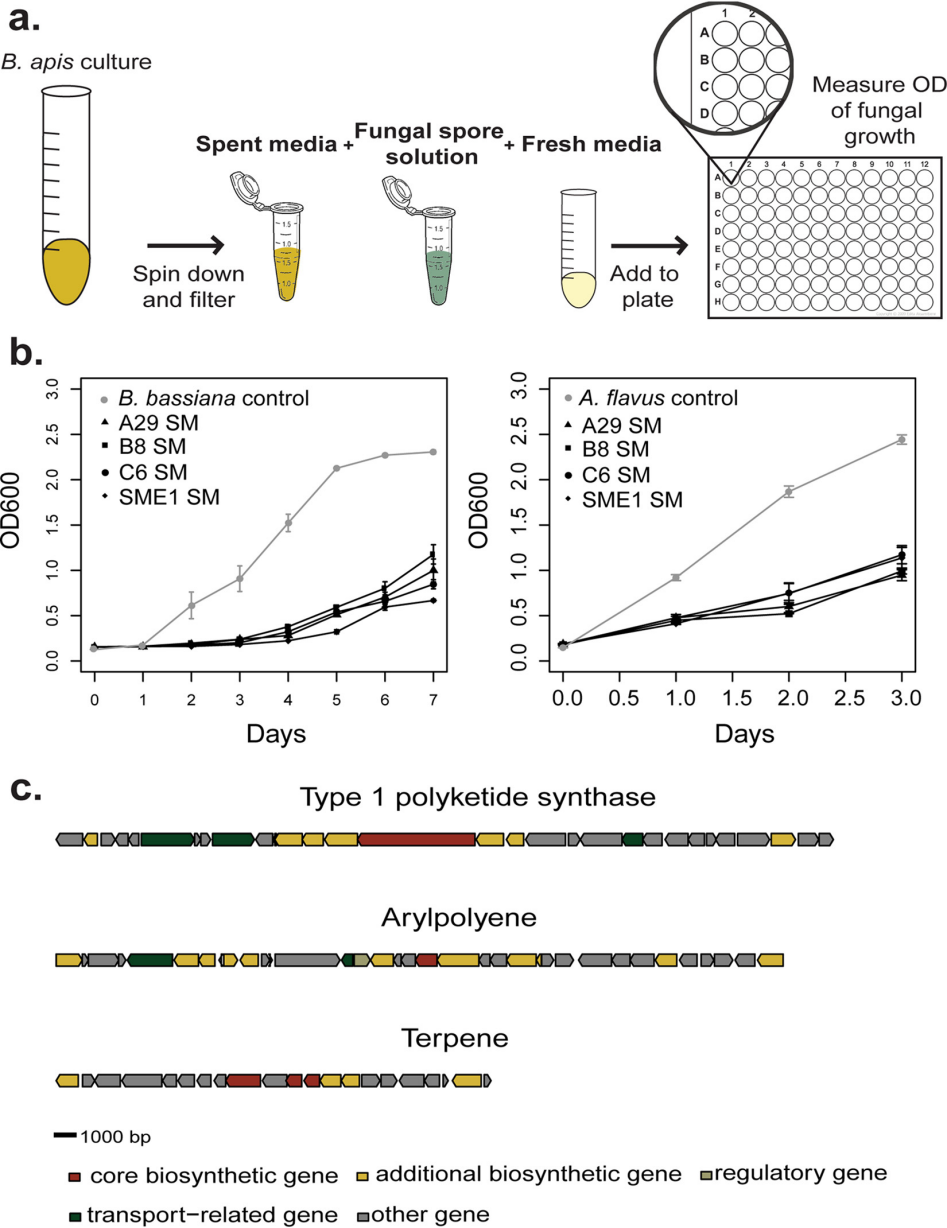
Fungal inhibition is mediated by *B. apis*-secreted metabolites. (a) Spores of fungal isolates were incubated in spent medium (SM) from *B. apis* cultures in addition to fresh medium or in fresh medium alone. (b) The growth of both B. bassiana (A29, *t* = −15.315, df = 119, *P* < 0.001; B8, *t* = −13.925, df = 119, *P* < 0.001; C6, *t* = −13.202, df = 119, *P* < 0.001; SME1, *t* = −11.963, df = 119, *P* < 0.001) and A. flavus (A29, *t* = −11.398, df = 59, *P* < 0.001; B8, *t* = −13.022, df = 59, *P* < 0.001; C6, *t* = −13.282, df = 59, *P* < 0.001; SME1, *t* = −11.261, df = 59, *P* < 0.001) in SM was strongly reduced compared to the control in fresh media, suggesting secreted metabolites from *B. apis* mediate fungal inhibition. The same volume of fresh media was in control and experimental wells. Significant inhibition via SM alone was observed in upwards of three independent experiments. (c) Genomic architecture of the terpene, type 1 polyketide synthase, and arylpolyene secondary metabolite gene clusters identified by antiSMASH; gene models are colored based on putative function within the cluster and are oriented to show direction of transcription.

We hypothesized that the mechanism by which *B. apis* protects the brood is secretion of an antifungal metabolite(s). Therefore, we incubated fungi in spent medium (SM) from *B. apis*, filtered to exclude bacterial cells. This spent medium would contain any metabolic by-products of *B. apis* metabolism, including secondary metabolites with antifungal properties. Fungal isolates were cultured in either fresh medium in addition to SM from *B. apis* cultures or in fresh medium alone. To normalize across symbiont strains, the cultures used to obtain the SM were diluted and normalized to the lowest optical density (Fig. 3a). Growth of both B. bassiana and A. flavus was significantly reduced by spent medium alone, indicating that *B. apis*-induced changes in the media are sufficient to suppress fungal growth. To eliminate the possibility that fungal inhibition was mediated by acidification of the media, A. flavus was cultured in media acidified to pH 5.0 (the same pH of *B. apis* SM). pH had no significant effect on fungal growth (Fig. S3; *t* = −0.251, df = 35, *P* = 0.804). Therefore, it is likely that *B. apis* inhibits fungi via secretion of an antifungal secondary metabolite(s). In search for possible loci that could contribute to the synthesis of the putative metabolite, we used antiSMASH (28) to annotate secondary metabolite gene clusters in the genomes of all *B. apis* strains used in this study. Interestingly, genomes of all strains encode a conserved type 1 polyketide synthase (T1PKS) gene cluster (Table S1). Type 1 polyketide synthases are common among host-associated microbes and produce macrolides that often have antifungal activity (15, 29–31). Additionally, all *B. apis* strains contain two biosynthetic gene clusters (BGCs) predicted to synthesize an aryl polyene and terpene.

10.1128/mBio.00503-21.3 FIG S3 Fungal inhibition by SM is not pH mediated. *B. apis* (A29) reduces MRS medium from pH 5.5 to 5.0. Spent medium from *B. apis* at pH 5.0 significantly reduced fungal growth (*t* = −6.111, df = 35, *P* < 0.001), while MRS medium reduced to pH 5.0 using HCl did not significantly reduce growth (*t* = −0.251, df = 35, *P* = 0.804). Download FIG S3, EPS file, 0.7 MB. Copyright © 2021 Miller et al. 2021 Miller et al. https://creativecommons.org/licenses/by/4.0/ This content is distributed under the terms of the Creative Commons Attribution 4.0 International license.

10.1128/mBio.00503-21.5 TABLE S1 Genome accessions for contigs from four *B. apis* strains harboring biosynthetic gene clusters. Download Table S1, DOCX file, 0.01 MB. Copyright © 2021 Miller et al. 2021 Miller et al. https://creativecommons.org/licenses/by/4.0/ This content is distributed under the terms of the Creative Commons Attribution 4.0 International license.

We performed two additional experiments to determine the composition of the antifungal. Treatment of the spent medium with either protease or heat did not diminish the antifungal effect (Fig. S4). This result suggests that the antifungal metabolite does not include a significant protein component. Further functional characterization of these gene clusters will help elucidate whether they play a role in the antifungal phenotype of *B. apis*. Considering the antifungal activity of *B. apis*-secreted metabolites *in vitro* and our genomic predictions, it is likely that *B. apis* synthesizes and secretes a metabolite capable of inhibiting fungi.

10.1128/mBio.00503-21.4 FIG S4 Protease and heat treatment do not reduce the antifungal activity of *B. apis* SM. Spent supernatant of a stationary *B. apis* culture was treated with proteinase K (a) or boiled for 15 min at 95°C (b) to denature proteins in the SM. Treated SM was assayed in fresh media with 10^3^ spores of B. bassiana to quantify its ability to inhibit fungal growth (measured by OD_600_). The increase in optical density of the heat-treated group is reflective of an increase in lysed bacterial cells, not fungal growth; there is no difference in the optical density of heat-treated SM alone versus B. bassiana incubated with heat-treated SM. Download FIG S4, EPS file, 0.7 MB. Copyright © 2021 Miller et al. 2021 Miller et al. https://creativecommons.org/licenses/by/4.0/ This content is distributed under the terms of the Creative Commons Attribution 4.0 International license.

## DISCUSSION

Fungi are significant and cosmopolitan pathogens of insects that can cause widespread and destructive damage. In eusocial insects, fungal infection can be especially problematic given how closely associated nestmates are and how quickly spores can spread within the hive. Here, we discovered that the honey bee has coopted the antifungal properties of a bacterial symbiont, Bombella apis, to protect its brood. Beyond decreasing brood losses and fungal load via direct inhibition of fungal infection, the presence of *B. apis* may also limit disease transmission by reducing the number of spores produced per infection. The bee would not be the first insect to use bacterial products to protect brood and other resources from fungal fouling. The Lagria beetles use *Burkholderia* antifungal metabolites to protect their brood (32), bee wolf larvae are protected by a *Streptomycetes* bacterial symbiont secreting an antifungal metabolite(s) (12, 45), and this same clade of actinobacteria is associated with ants and used to protect brood and other resources from invading fungi (33, 46). However, the production of an antifungal by an acetic acid microbe is a novelty. Therefore, it is possible that this antifungal metabolite(s) is a new structure or has a novel fungal target.

Using the antiSMASH (antibiotic and Secondary Metabolite Analysis Shell) pipeline, we identified three biosynthetic gene clusters (BGCs) in the genome of each *B. apis* strain sequenced (see Table S1 in the supplemental material). The first cluster product, an aryl polene, is unlikely to have any antimicrobial activity and is likely transported to the cell membrane. Similarly, the second gene cluster product, a terpene, is likely a component of the *B. apis* membrane. Terpenes are produced by a wide variety of bacteria (34), but in many cases their structures are challenging to predict from genomics alone due to the promiscuity of core enzymes, such as terpene cyclases, involved in their biosynthesis (35). Within *Alphaproteobacteria*, the synthesis of pentacyclic triterpenoids, hopanoids, is common, and experimental validation in Rhodopseudomonas palustris identifies their involvement in stress response and membrane integrity (36–39). The annotated terpene biosynthesis cluster in the genome of *B. apis* bears some homology to a hopanoid BGC, evidenced by *hpnC* and *hpnD*, which are involved in the biosynthesis of the hopanoid precursor squalene (40). However, the incompleteness of this pathway in the *B. apis* genome paired with the low sequence similarity to hopanoid biosynthetic genes in R. palustris impedes any meaningful conclusions about the terpene’s structure or function.

In addition to these two BGCs, *B. apis* has a type 1 polyketide synthase gene cluster (T1PKS), a common BGC implicated in antifungal production by diverse bacterial strains. In addition to the core ketosynthase, acyl-transferase, and acyl carrier protein domains, the T1PKS annotation includes dehydrogenase, enoyl reductase, and ketoreductase domains, which further alter the structure of the core product and may alter activity (Fig. 3). This BGC is also predicted to be a PKS/NRPS hybrid, since it includes two putative adenylation domains. The predicted product from the TIPKS cluster bears low similarity to characterized polyketides and, therefore, may have a novel structure.

In addition to the antifungal candidates listed above, it is possible that an unannotated gene cluster is responsible for antifungal production. Because the MIBiG database for secondary metabolite clusters is biased toward *Actinobacteria* and *Streptomycetes* in particular, it is likely that genome annotation by antiSMASH is underpredicting the number of BGCs present in *B. apis* or even misannotating clusters. Given the phylogenetic distance between proteobacteria and the actinomycetes, it is probable that annotation with antiSMASH does not reflect the true biosynthetic capabilities of *B. apis*. Indeed, the predicted biosynthetic regions bear low similarity to known biosynthetic gene clusters (aryl polyene, 14% to xanthomonadin; terpene, 7% to malleobactins A to D; T1PKS, no noted similarities).

Our experiments conclusively show that *B. apis* secretes an antifungal metabolite that can protect bees from fungal infection. However, whether *B. apis* load in the colony is correlated with fungal disease prevalence in broods has not been explored. One previously published field experiment supplemented *B. apis* in the pollen patties provided to honey bee colonies and observed a reduction in *Nosema* load in adult bees (23), perhaps due to interactions with *B. apis* in larvae, which may serve as temporary reservoirs for *Nosema* (41), or through indirect effects on honey bee health via nutrition. Whether this correlation between *B. apis* and *Nosema* load is direct is unclear, and addressing this relationship would necessitate *in vitro* assays. Unfortunately, since *Nosema* cannot currently be cultured outside the host, it was not included in our *in vitro* competition and SM assays. Further assays of phylogenetically distant fungal species coupled with identification of the method of action by which *B. apis* inhibits fungal growth will determine if *B. apis* is an effective antifungal treatment for diverse fungi, including *Nosema*.

Altering the prevalence of pathogenic fungi within managed honey bee colonies could have ecological consequences beyond the honey bee. Floral resources shared among diverse pollinators act as transmission centers for fungi, both pathogenic and saprophytic (42). Species-specific fungal pathogens can be seeded in pollen and nectar sources (43), after which diverse pollinators, including native bees, can act as vectors to transmit the fungal pathogens to other floral sources, thereby facilitating heterospecific transmission of fungal agents (44). As a result of reduced spore loads within colonies, the load of fungal pathogens deposited in local floral resources by foragers might also decrease and perhaps reduce heterospecific transmission and spillover events (44).

## MATERIALS AND METHODS

### Isolates and culturing.

All bacterial strains of *B. apis* were obtained by sampling either nectar or larvae (Table 1). Isolates were acquired from our apiary or from Leibniz-Institut DSMZ. All cultures were incubated for 48 h at 34°C in MRS (de Man, Rogosa, and Sharpe). Fungal isolates B. bassiana and A. flavus were maintained at 25°C with 80% relative humidity or 34°C with ambient humidity, respectively, on PDA (potato dextrose agar) or MRS agar plates. All media were purchased from Fisher Scientific. Spore solutions were prepared by flooding fungal plates with 0.01% Triton X-100, agitating with a cell scraper, and suspending the spores in the solution.

**TABLE 1:** Sampling of *B. apis* strains

Species	Strain	Origin	Sample	Genome GenBank accession no.
*B. apis*	SME1	IN	Nectar	GCA_009362775.1
*B. apis*	A29	AZ	Larvae	GCA_002917995.1
*B. apis*	B8	AZ	Larvae	GCA_002917945.1
*B. apis*	C6	AZ	Larvae	GCA_002917985.1

### Competition plates.

*B. apis* strains were grown to their maximal optical density (OD), and all strains were normalized to the lowest OD value by diluting in fresh media. A lawn of *B. apis* was created by plating 100 μl of normalized culture on MRS agar plates. The plate was then inoculated with 10^3^ spores of each fungal isolate and incubated at the appropriate temperature for that isolate (34°C for A. flavus and 28°C for B. bassiana). Over the course of 4 to 7 days (four for A. flavus and seven for B. bassiana), the presence of hyphal/conidial growth was monitored.

### Competition assays.

*B. apis* strains were grown to their maximal OD, and all strains were normalized to the lowest OD value by diluting in fresh media. A total of 10^3^ spores of each fungal isolate were incubated in 100 μl of density-normalized *B. apis* culture and 100 μl fresh media. Fungal growth was monitored daily, and once control plates of fungus cultured without the symbiont showed sporulation, spore counts were quantified for each well via hemocytometer.

### Larval collection and *in vivo* infections.

Late first instars were grafted from our apiary at the Indiana University Research and Teaching Preserve into queen cups filled with UV-sterilized worker diet prepared as outlined by Schmehl et al. (47). *B. apis*-supplemented groups were given a diet with a ratio of 1:4 stationary (OD, 1.0) *B. apis* in MRS to worker diet. This bacterial load was between 2 × 10^6^ and 6 × 10^6^ cells/ml. Control groups were given diet with a ratio of 1:4 axenic MRS media to worker diet. After 5 days in larval diet, prepupae were transferred to new wells after either MRS or *B. apis* in MRS was added. Five days into pupal development, individuals were inoculated with 10^3^ spores of A. flavus in 0.01% Triton X-100 or an equal volume of 0.01% Triton X-100 as a control. *B. apis*-supplemented groups were coinoculated with a final dose of the bacterium (10^4^ cells); controls received the same volume of MRS. The final dose of *B. apis* was administered to ensure that metabolites produced by the bacterium were still present at the time of fungal inoculation. To assess if the bacterium and its produced metabolites are still present and active 5 days into the pupal stage, further experiments would have to be performed. The pupal stage was chosen as the point of fungal inoculation for two reasons. (i) The survival rate of laboratory-reared larvae to adulthood is low, and the likelihood of survival is lower during the larval stage. Performing this infection assay in pupae reduces the noise created by stochastic death of individual replicates. (ii) To minimize variation between replicates, all broods are age matched by eye. During pupation, the melanization of the developing eyes serves as a standard by which broods can be age matched. The presence of infections (as evidenced by hyphae penetrating through the cuticle and/or spore production) was scored daily until adulthood. Although the data in this paper reflect the results of one experiment, the same results were replicated in two separate experiments, including with a different *B. apis* strain (Fig. S2).

### Analysis of BGCs.

Genomes for all strains were downloaded from GenBank (see Table 1 for accession numbers) and reannotated with RAST (43, 44). The resulting GFF files and corresponding genome files were uploaded to antiSMASH (28), and results were compared across strains to determine conserved secondary metabolite synthesis clusters. Gene model figures were visualized and adapted for publication using R.

### *In vitro* antifungal assay.

To obtain spent medium, strains were grown to their maximal OD (0.6 to 0.25), and all strains were normalized to the lowest OD value by diluting in fresh media. Cultures were spun down at 7,600 relative centrifugal force for 5 min, and the supernatant was filtered through a 0.2-μm filter to remove bacterial cells. Spent medium and fresh medium were added to a multiwell plate in equal volumes, and 10^3^ spores from spore stock solutions were added. Growth was measured daily by assaying OD at 600 nm (OD_600_). A positive control included spores in fresh media alone, used to compare to treatment groups with spent medium. Optical densities of spent medium alone were monitored to ensure no bacterial growth occurred. Assay plates were incubated at the appropriate temperature for the fungal isolate used (34°C for A. flavus and 28°C for B. bassiana). Since *B. apis* acidifies the media from pH 5.5 to 5.0, controls of MRS medium reduced to pH 5.0 with HCl were included (Fig. S3).

### Statistical analyses.

All statistical analyses were performed in R version 3.5.1. Spore counts from fungi cultured in MRS medium alone (controls) were compared to spore counts from fungi cultured in the same volume of MRS medium as the controls in addition to an equal volume of stationary *B. apis* culture (A29, B8, C6, and SME1). Nonparametric tests [wilcox.test(), kruskal.test()] or parametric tests [*t* test()] were used to compare across the data set and in pairwise comparisons between spores in fresh media without the bacterial symbiont to spores cocultured with the symbiont; *P* values were Bonferroni corrected for multiple comparisons across strains. *In vivo* infections are displayed as Kaplan-Meier survival curves. Treatments with or without *B. apis* infection were compared with a log-rank test using R package version 3.5.1, “survminer.” Interactive effects of *B. apis* SM on growth of fungi over time were determined with a generalized linear model of OD, time, and strain identity.
