# Supplementary material for: A Bacterial Symbiont Protects Honey Bees from Fungal Disease
Source: mBio. 2021 Jun 8;12(3):e00503-21. doi: 10.1128/mBio.00503-21 (PMC8262860; doi:10.1128/mBio.00503-21)
Supplement: TABLE S1 [file mbio.00503-21-st001.docx]

**Supplemental Table 1:** Genome accessions for contigs from four *B. apis* strains harboring biosynthetic gene clusters.

| Genome accession | Strain | TIPKS | Aryl polyene | Terpene |
| --- | --- | --- | --- | --- |
| LMYH00000000 | A29 | **NZ_LMYH01000013** | **NZ_LMYH01000016** | **NZ_LMYH01000009** |
| LMYI00000000 | B8 | **NZ_LMYI01000001** | **NZ_LMYI01000015** | **NZ_LMYI01000007** |
| LMYJ00000000 | C6 | **NZ_LMYJ01000002** | **NZ_LMYJ01000010** | **NZ_LMYJ01000007** |
| NZ_WHNS00000000 | SME1 | **NZ_WHNS01000005** | **NZ_WHNS01000003** | **NZ_WHNS01000010** |
